# CSF2RB Is a Unique Biomarker and Correlated With Immune Infiltrates in Lung Adenocarcinoma

**DOI:** 10.3389/fonc.2022.822849

**Published:** 2022-04-28

**Authors:** Ningning Zhu, Yueyang Yang, Haitong Wang, Peng Tang, Hongdian Zhang, Haiyan Sun, Lei Gong, Zhentao Yu

**Affiliations:** ^1^ Department of Esophageal Cancer, Tianjin Medical University Cancer Institute and Hospital; National, Clinical Research Center for Cancer, Key Laboratory of Cancer Prevention and Therapy, Tianjin’s Clinical Research Center for Cancer, Tianjin, China; ^2^ Department of Thoracic Surgery, National Cancer Center, National Clinical Research Center for Cancer, Cancer Hospital & Shenzhen Hospital, Chinese Academy of Medical Sciences and PeKing Union Medical College, Shenzhen, China

**Keywords:** CSF2RB, immune infiltrate, tumor microenvironment, biomarker, tumor-suppressor gene

## Abstract

**Background:**

The tumor microenvironment plays an important role in the occurrence and development of tumors. However, there are gaps in understanding the molecular and cellular interactions between tumor cells and the immune tumor microenvironment (TME). The aim of this study was to identify a novel gene that played an important role in the tumor microenvironment of lung adenocarcinoma (LUAD).

**Methods:**

The gene expression profile and clinical data for LUAD were downloaded from TCGA database. First, we used the ESTIMATE algorithm to evaluate the immune and stromal scores accordingly. Also, we analyzed differentially expressed immune-related genes (IRGs) in the high and low immune/stromal score groups. Then, we used the protein–protein interaction network (PPI network) and a univariate Cox regression analysis to identify the hub gene. After that, we analyzed the relationship between CSF2RB expression and TNM stage/prognosis. Furthermore, gene set enrichment analysis (GSEA) was used to analyze the pathway regulated by CSF2RB and the Pearson correlation analysis method was used to analyze the correlation between the CSF2RB and immune cells. Finally, we used Western blot, real-time quantitative PCR (RT-qPCR), and immunohistochemistry (IHC) to validate CSF2RB expression in cancer and para-cancerous tissues.

**Results:**

We identified that CSF2RB played an important role in the tumor microenvironment of LUAD. The expression of CSF2RB in tumor tissues was lower than that in normal tissues. Furthermore, the Kaplan–Meier plotter showed that a low CSF2RB expression was associated with poor survival and multivariate COX regression analysis revealed that the CSF2RB gene was an independent risk factor for prognosis, independent of whether patients received chemotherapy or radiotherapy. More importantly, a high expression of CSF2RB was related to early T, N, and clinical stages. GSEA analysis revealed that CSF2RB associated with diverse immune-related pathways, including T-cell receptor signaling pathway, Toll-like receptor signaling pathway, and B-cell receptor signaling pathway. CSF2RB expression levels were also positively related with the levels of infiltrating CD4+ T cells, macrophages, NK cells, and monocytes in LUAD. Finally, tumor tissues from LUAD patients were used for the assessment of CSF2RB expression. It was significantly lower in tumor sites than in adjacent normal tissues, which was consistent with data analysis.

**Conclusion:**

CSF2RB effectively predicted the prognosis of patients with lung adenocarcinoma which could also be a potential target for cancer treatment and prevention. However, further studies are required to elucidate the function and regulatory mechanisms of CSF2RB and to develop some novel treatment strategies.

## Introduction

Lung cancer is considered the major cause of cancer deaths in the world, with more than 1.6 million new cases diagnosed every year (1). Lung adenocarcinoma (LUAD) is the major subtype of lung cancer (2). However, the precise molecular mechanisms relevant to LUAD development and progression remains to be unclear. In recent years, immunotherapy has achieved a breakthrough by increasing the survival rates for NSCLC patients (3). However, cancer cells promoted T-cell exclusion and resistance to checkpoint blockade, which still resulted in poor prognosis (3–5). More and more evidence has shown that the characteristics of TME play a crucial role in mediating late metastasis, immune escape, and immunotherapy resistance (6–8). In recent years, a growing number of research works have been published in the field of cancer immunotherapy, which related to TME. There is a strong tumor cell protection network, which helps tumor cells to fight against the immune system, and it enables tumor cells to escape the surveillance of immune cells. The components of the TME include immune cells, mesenchymal cells, endothelial cells, inflammatory mediators, and extracellular matrix molecules (9). Tumor-infiltrating immune cells (TIICs) and stromal cells are the two main non-tumor cell components, which have been considered valuable for tumor diagnosis and prognostic evaluation (10). Studies have shown that the malignancy of a tumor depends not only on the features of the tumor cells but also on the components of the environment surrounding the tumor (11–13). Studies have shown that the predictive value of tumor-infiltrating lymphocytes (TILs) in the tumor microenvironment of lung adenocarcinoma and lung squamous cell carcinoma has statistical significance (14). Cytotoxic T cells, memory T cells, and T-helper type 1 cells were positively correlated with good prognosis in breast cancer, ovarian cancer, colorectal cancer, lung cancer, and beyond. High densities of CD3+ T cells, CD8+ cytotoxic T cells, and CD45RO+ memory T cells were linked to longer disease-free survival and/or overall survival (15–17). In addition, it was reported that the expression level of eight Th1/IFNγ-related genes in tumor-positive lymph node tissue predicted the duration of disease-free survival (DFS) and overall survival (OS) in melanoma (18). These findings clarified the link between TME and cancer progression, and prospective therapy options to improve the treatment of malignant tumors.

Colony-stimulating factor 2 receptor beta (CSF2RB) is on chromosome 22 and encodes the β chain of the GM-CSF receptor. It is the shared subunit βc of interleukin 3 (IL3), GM-CSF, and the IL5 receptor. The ligand-specific alpha chain (CSF2RA) forms a dodecameric complex with CSF2RB and the GM-CSF ligand during GM-CSF signaling, in which the closeness of CSF2RB subunits permits related JAK2 kinases to trans-phosphorylate (19). It mainly promotes survival, proliferation, and differentiation through the JAK2/STAT5, PI3K/mTOR, and MEK/ERK pathways (20). A germline mutation in the CSF2RB gene from a T-cell acute lymphoblastic leukemia (T-ALL) patient was found to stimulate factor-independent cell proliferation *in vitro*, according to a study. They demonstrated that a targetable CSF2RB variation present in human leukemia promotes factor-independent growth, receptor phosphorylation and accumulation, and JAK/STAT pathway activation (21).

Although several immune-related genes in LUAD have been identified in recent years, the mechanisms of immune cell infiltration in the TME mediated by key genes are still to be explored. The aim of this study was to identify a novel gene that played an important role in the tumor microenvironment of LUAD.

## Materials and Methods

### Data Source and Preprocessing

515 cases of gene expression profiles (including 515 adenocarcinoma and 59 matched para-carcinoma tissues) and clinical information of LUAD were downloaded from TCGA database. Clinical information included gender, age, tumor grade, clinical stage, and survival time. Perl software was used for data collation and integration to obtain gene expression matrix and clinical data. Analysis was subsequently carried out.

### Microenvironment Score

We normalized the matrix data of gene expression by using the LIMMA package of R software (version 3.6.2). Then, the ESTEATE estimation algorithm was applied to the matrix data to calculate the immune and stromal scores. In addition, according to the median immune and stromal scores, OS cases were divided into the high immune score group and low immune score group to determine the relationship between immune score and overall survival rate.

### Correlation Analysis of Microenvironment Score/Hub Gene and Survival Rate/Clinical Characteristics

Survminer is a survival analysis tool that was used in R language. The survival curve was drawn by the Kaplan–Meier method, and the log-rank sum was tested for statistical significance, p < 0.05. LIMMA and ggplot2 packages were used to analyze the relationship between microenvironment score/hub gene and clinical characteristics.

### Gene Differential Expression Analysis

Expression differences of genes were calculated with the LIMMA R package, setting fold change >1 or <-1, p-value <0.05 as limit. The heat map is generated using the pheatmap package in the R software.

### GO and KEGG Enrichment Analysis

GO and KEGG enrichment analyses were performed on 379 DEGs with the help of clusterProfiler, enrichplot, and ggplot2 R packages. Both p-value and q-value of <0.05 were regarded as significantly enriched.

### Protein–Protein Interaction Network Construction, Hub Genes, and Module Analysis

The STRING database was conducted to identify the probable links among these overlapping DEGs at the protein level with a combined score >0.4. The PPI network was rebuilt in Cytoscape v3.7.2.

### Survival Outcome and Multivariate/Univariate Cox Analysis

Univariate Cox regression analysis was used to determine the relationship between DEG expression level and patient OS; the graph shows genes with a p-value <0.05. Multivariate cox regression analysis was used to analyze the correlation between multiple factors and survival rate.

### Gene Set Enrichment Analysis

Gene Set Enrichment Analysis (GSEA) was performed on the entire transcriptome of all tumor samples, and only gene sets with NOM p < 0.05 and FDR q < 0.05 were considered significant.

### Inference of the Abundance of Infiltrating Immune Cells in TME

We used the CIBERSORT algorithm (16) and the LM22 gene signature to quantify the proportion of immune cells in LUAD samples. CIBERSORT is a deconvolution algorithm that employs a set of gene expression reference standards (with signatures of 547 genes) that are considered to be the smallest representation of each cell type. It employs linear support vector regression (SVR) to deconvolute a mixture of gene expression.. A vast number of tumor samples were used to infer the fraction of cell types in the data. Gene expression profiles were prepared using standard annotation files, and the data were uploaded to the CIBERSORT web portal (http://cibersort.stanford.edu/). The technique uses 1,000 permutations and LM22 signatures.

### Correlation Analysis Between Immune Cells and Hub Gene

We employed the Pearson correlation analysis approach to analyze the correlation between the hub gene and 22 immune cells which were obtained by CIBERSORT on the slices to identify immune cells related to survival. The canvasXpress R package visualizes the correlation index r and the related p value.

### Human Specimens

Human samples (34 lung adenocarcinoma tissue and 34 para-cancerous tissues) were collected from the Tianjin Medical University Cancer Institute and Hospital. All selected patients received surgery without neoadjuvant therapy, such as chemotherapy, radiotherapy, and immunotherapy. Cancer tissues and para-cancerous tissues selected for real-time quantitative PCR (RT-qPCR), WB, and immunohistochemical (IHC) experiments were paired in the same patient. Para-cancerous tissue was taken from samples 5 cm away from the cancer tissue. Informed consent was provided by all participants, and the protocol was approved by the Human Ethics Committee of Tianjin Medical University (No. bc2021177). The clinical data of patients can be obtained in the **Supplementary Materials**.

### Real−Time Quantitative PCR Analysis

To determine whether differences exist in the expression levels of CSF2RB between lung adenocarcinoma tissues and para-cancerous tissues, we collected 14 lung adenocarcinoma tissue specimens. The procedure was approved by Tianjin Medical University’s Human Ethics Committee, and all subjects gave written informed permission. The total RNA of the cells was extracted by TRIzol Reagent (Invitrogen, Carlsbad, CA, USA) and then was used to synthesize cDNA using PrimeScript RT Reagent Kit (TaKaRa Bio, Shiga, Japan) following the manufacturer’s instruction. RT-qPCR was carried out to estimate the expression of CSF2RB and the mRNA levels of CSF2RB with SYBR Green I Master Mix Kit (Invitrogen, Carlsbad, CA, USA) and 7300 Real-Time PCR System (Applied Biosystems, Foster City, CA, USA). GAPDH was used as internal control for CSF2RB. The relative expression value was calculated using the 2^−ΔΔ t^ method and normalized to the endogenous control gene. The paired-sample T test was applied to test the differential expression of CSF2RB in cancer tissues compared to adjacent non-cancerous tissues.

### Immunohistochemical Staining of CSF2RB

LUAD tissues were purchased from the Tumor Tissue Bank of the Tianjin Cancer Hospital, which contained 20 carcinoma tissues and paired para-carcinoma tissues. Tissues were incubated using rabbit polyclonal anti-CSF2RB antibody (1:100 dilution; catalog no. ab52609) overnight at 4°C. Using the double-blind method, two senior pathologists evaluated all the staining results without knowing the clinical information and prognosis of all patients.

### Western Blotting

We collected 6 lung adenocarcinoma specimens from Tianjin Cancer Hospital and obtained informed consent from the patients. The lung adenocarcinoma tissue was lysed with a cell lysate. Sodium dodecyl sulfate-polyacrylamide gel electrophoresis was used to separate proteins of different molecular weights and then transfer them to nitrocellulose membranes. Membranes were blocked with 5% non-fat milk for 1.5 h and then at 4°C with GAPDH primary antibody (1:1,000, Zhongshan Jinqiao, Beijing, China) and CSF2RB primary antibody (1:1,000, Abcam, Cambridge, UK) and incubated overnight in the primary antibody dilution. After that, the membrane was rinsed with TBST and incubated for 1.5 h with the secondary antibody. Each band was detected using the ECL Color Development kit (Thermo Fisher, Scotts Valley, CA).

### Statistical Analysis

R v3.6.2 and IBM SPSS 25.0 were used to conduct basic statistical analysis. For non-parametric data, the Wilcoxon test and Kruskal–Wallis H test were used to establish significance; a p-value < 0.05 was considered to be statistically significant, with the exception of correlation analysis. The cor.test package in R was used to calculate Pearson correlations, and the ggpubr and corrplot packages in R were used to create correlation graphs. Only p values less than 0.0001 were considered to be statistically significant for correlations, and correlation strength was classified as follows: 0.00–0.19 “very weak,” 0.20–0.39 “weak,” 0.40–0.59 “moderate,” 0.60–0.79 “strong,” and 0.80–1.0 “extremely strong.” Kaplan–Meier and Cox regression analyses were performed and visualized using the survival and survminer packages in R. The function “surv cutpoint” from the “maxstat” R package was used to produce the best cutpoint for these studies.

## Results

### The Effects of Immune and Interstitial Scores on Progression and Prognosis of Lung Adenocarcinoma Patients

We downloaded TCGA-derived gene expression profiles and clinical information of LUAD. A total of 515 patients were obtained, including 515 adenocarcinoma and 59 matched para-carcinoma tissues. Among the enrolled patients, 256 (49.71%) were older than 65, and 238 (46.21%) were below 65. There were 235 men (45.63%) and 271 women (52.62%). 3 patients (0.58%) received neoadjuvant chemotherapy. 175 patients (33.98%) received postoperative chemotherapy, and 98 patients (19.03%) received postoperative radiotherapy. According to the American Joint Committee on Cancer (AJCC) 8th edition staging manual for lung cancer, this study included 270 patients (52.43%) at stage I, 119 patients (23.11%) at stage II, 81 patients (15.73%) at stage III, and 26 patients (5.05%) at stage IV (**Table 1**). The clinical information of the patients is shown in **Table 1**.

**Table 1:** Clinical and pathological characteristics.

Factor	N% or median (range)
Sex	
Male	235 (45.63%)
Female	271 (52.62%)
Data missing	9 (1.75%)
Age	
>65	256 (49.71%)
≤65	238 (46.21%)
Data missing	21 (4.08%)
Chemotherapy	
Yes	175 (33.98%)
No	329 (63.88%)
Radiotherapy	
Yes	98 (19.03%)
No	406 (78.83%)
Neoadjuvant therapy	
Yes	3 (0.58%)
No	500 (97.09%)
Data missing	1 (0.19%)
Path T-stage	
T1	168 (32.62%)
T2	269 (52.23%)
T3	45 (8.74%)
T4	19 (3.69%)
Data missing	14 (2.72%)
Path N-stage	
N0	325 (63.11%)
N1	94 (18.25%)
N2	71 (13.79%)
N3	2 (0.39%)
Data missing	23 (4.47%)
Path M-stage	
M0	335 (65.05%)
M1	25 (4.85%)
Data missing	155 (30.10%)
Clinical stage	
I	270 (52.43%)
II	119 (23.11%)
III	81 (15.73%)
IV	26 (5.05%)
Data missing	19 (3.69%)
Survival status	
Alive	321 (62.33%)
Dead	183 (35.53%)
Data missing	11 (2.14%)

**Figure 1:**
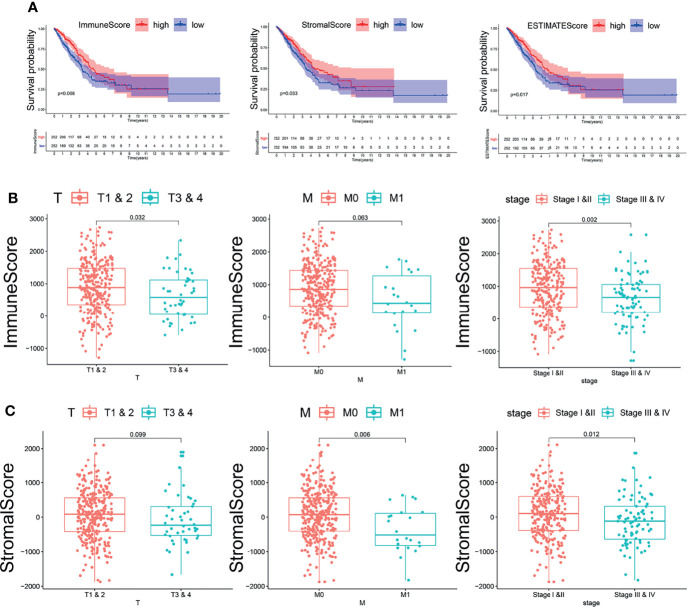
The relationship between immune and stromal scores and clinical features and prognosis of lung adenocarcinoma. **(A)** The relationship between immune and stromal scores and prognosis of lung adenocarcinoma. The red line represents the high-scoring group, and the blue line represents the low-scoring group. **(B)** The correlation between immune scores and clinicopathological features. **(C)** The correlation between stromal scores and clinicopathological features.

According to the ESTIMATE algorithm, the interstitial scores of LUAD patients were -1,871.27~2,102.348 points; the immune scores of LUAD patients were -1,285.37~2,733.237 points (**Supplementary Table 1**). Kaplan–Meier survival analysis and log-rank test were used to determine the potential correlations between overall survival (OS) and immune and stromal scores. The Kaplan–Meier plot demonstrated that patients with high immune scores and stromal scores had better clinical outcomes (p < 0.05, **Figure 1A**). The correlation between immune scores and stromal scores with clinicopathological features was then further analyzed. It showed that the stromal score and immune score from the human samples at the age >65 and female groups were notably higher than those at the age ≤65 and male groups (**Supplementary Figures 1A, B, D, E**, p < 0.05). Moreover, higher immune scores were correlated with lower T stage (T1–2). Meanwhile, high stromal scores were correlated with early M stage (M0). Based on the clinical stage classification, both the stromal scores and immune scores were significantly higher in early stages (I–II) than advanced clinical stages (III–IV). While the stromal scores and immune scores showed no correlation with the other pathologic stages, they showed the same tendency (**Figures 1B, C** **)**. These results indicated that the stromal scores and immune scores might indicate the progression of LUAD.

To better understand the impact of immune and stromal characteristics on LUAD, we used the CIBERSORT algorithm to assess the infiltration ratio of 22 immune cells (TIICs) in LUAD patients (**Supplementary Figure 2A**). The TIICs are mainly related to adaptive immunity [that is, memory B cells, naive B cells, activated memory CD4 T cells, resting memory CD4 T cells, naive CD4 T cells, CD8 T cells, T follicular helper cells (Tfh), γδ T cells (TGD), and regulatory T (Treg) cells] and natural immunity [i.e., activated dendritic cells (DC), resting DC, eosinophils, macrophages (M0–M2)]. As shown in **Supplementary Figure 2B**, the correlation between the different TIICs of LUAD ranged from weak to moderate. When we used LIMMA and ggplot2 packages to analyze the relationship between TIICs and TNM staging, it showed that the infiltrating proportion of activated dendritic cells and plasma cells was high in patients younger than 65 years, the infiltrating proportion of M1 macrophages was high in patients older than 65 years, the infiltrating proportion of plasma cells was high in male patients, the infiltrating proportion of CD4 T memory resting cells was high in female patients, and the proportion of CD8+T cells in the N0–N1 stages were higher than in the N2–N3 stages. Moreover, neutrophils in stages III–IV were higher than those in stages I–II. Activated dendritic cells and plasma cells infiltrate a high proportion of patients younger than 65 years (**Supplementary Figure 3A**, p < 0.05). Kaplan–Meier analysis showed that the lower the infiltrating proportion of activated mast cells and Treg cells and the higher the infiltrating proportion of monocytes, the higher the survival rate of patients (**Supplementary Figure 3B**, p < 0.05). These results indicated that in the stromal and immune components of the tumor microenvironment, Treg cells, CD8+T cells, CD4 T memory resting cells, plasma cells, activated mast cells, activated dendritic cells, and M1 macrophages played an important role in the development of LUAD.

### Gene Expression Analysis and Functional Enrichment Analysis of LUAD Patients

**Figure 2:**
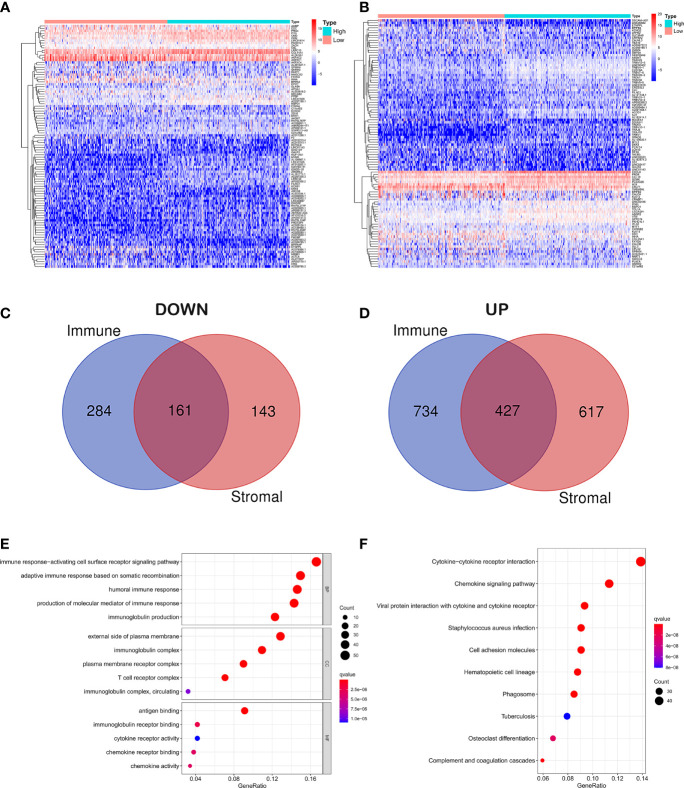
Differential gene expression analysis and enrichment analysis. **(A)** Differential analysis of stromal cell components. **(B)** Differential analysis of immune cell components. **(C)** Overlapping genes that were downregulated in the immune group and the interstitial group. **(D)** Overlapping genes that were upregulated in the immune group and the interstitial group. **(E)** GO analysis of DEGs classified DEGs into three functional groups: CC, BP, and MF group. **(F)** Signaling pathway analysis of KEGG.

In order to reveal gene expression profile changes in different stroma and immune scores, we used the LIMMA R package to analyze gene expression data obtained from 515 cases of LUAD in TCGA. According to the estimated scores, all samples were divided into high/low immune score groups and high/low stromal score groups, and cross genes were screened. The heat map which was generated using the pheatmap package showed the different gene expression profiles from the cases according to high or low immune/stroma score groups (**Figures 2A, B** **)**. In the high immune score group, there were 1,161 upregulated genes and 445 downregulated genes (fold change >1, p < 0.05). Similarly, in the high stroma score group, 1,044 genes were upregulated and 304 genes were downregulated (fold change >1, p < 0.05). As shown in the Venn diagram (**Figures 2C, D** **)**, we selected overlapping genes that were upregulated or downregulated in the immune group and the interstitial group as immune-related genes (427 upregulated genes and 161 downregulated genes). These overlapping genes were selected for all subsequent analyses in this study.

In order to explore the potential molecular mechanism of immune-related genes, we used clusterProfiler, enrichplot, and ggplot2 packages to perform functional enrichment analysis. GO analysis of differentially expressed genes (DEGs) was classified into three functional groups: biological process (BP), cellular component (CC), and molecular function (MF) groups. Results indicated that the overlapping DEGs in BP of GO enrichment were markedly associated with immune response-activating cell surface receptor signaling pathway, adaptive immune response based on somatic recombination of immune receptors built from immunoglobulin superfamily domains, humoral immune response, production of molecular mediator of immune response, and immunoglobulin production. As for CC, the overlapping DEGs were particularly enriched in the external side of the plasma membrane, immunoglobulin complex, plasma membrane receptor complex, T-cell receptor complex, immunoglobulin complex, and circulating. In addition to MF of GO enrichment, DEGs were remarkably related to antigen binding, immunoglobulin receptor binding, cytokine receptor activity, chemokine receptor binding, chemokine activity, and immunoglobulin receptor binding (**Figure 2E**). Besides, signaling pathway analysis of KEGG demonstrated that those DEGs played pivotal roles in cytokine–cytokine receptor interaction, chemokine signaling pathway, viral protein interaction with cytokine and cytokine receptor, staphylococcus aureus infection, cell adhesion molecules, hematopoietic cell lineage, phagosome, tuberculosis, osteoclast differentiation, and complement and coagulation cascades (**Figure 2F**).

### Intersection Analysis of Protein–Protein Interaction Network and Univariate COX Regression Analysis

**Figure 3:**
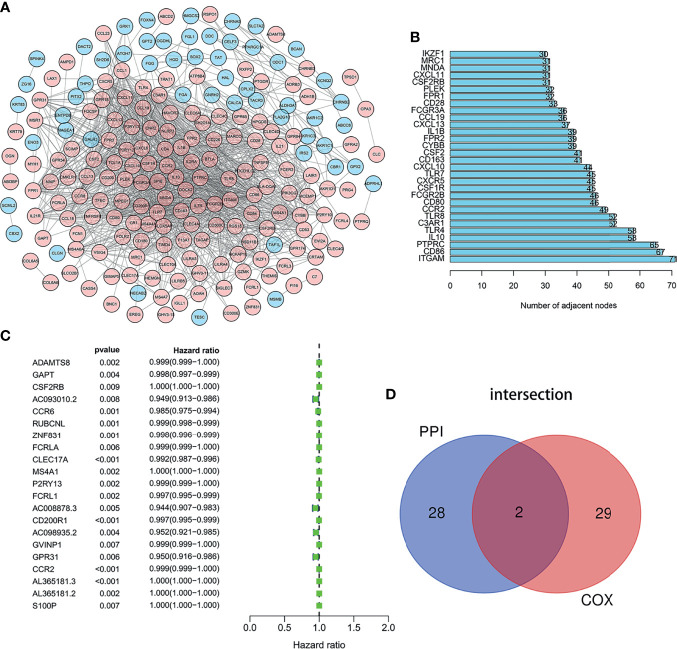
Intersection analysis of PPI network and univariate COX regression analysis. **(A)** The interaction between IRGs. **(B)** Top 30 genes most connected to other genes. **(C)** Univariate Cox regression analysis was performed on the survival of 379 LUAD patients to determine the significant genes affecting the survival of LUAD patients. **(D)** the intersection analysis between the leading nodes in the PPI network and the top 16 genes in the p-value in the univariate Cox regression.

We employed the STRING database and Cytoscape software to construct the PPI network, which included 312 nodes and 3,016 edges, to explore the interaction between IRGs (**Figure 3A**). Then 30 genes were selected for further analysis, such as ITGAM, CD86, PTPRC, IL10, TLR4, C3AR1, TLR8, CCR2, CD80, FCGR2B, and CSF2RB. These genes were highly interactive with all the other genes (**Figure 3B**). Univariate Cox regression analysis was performed on the survival of 379 LUAD patients to determine the significant factors affecting the survival of LUAD patients. The p-value of all the candidate genes was below 0.01, which is shown in **Figure 3C**. Then, the intersection analysis was performed between the leading nodes in the PPI network and in the univariate Cox regression. Only C–C motif chemokine receptor 2 (CCR2) and colony-stimulating factor 2 receptor subunit beta (CSF2RB) were found to overlap (**Figure 3D**).

### The Relationship Between CSF2RB Gene Expression Level and Survival Outcome/Clinicopathological Stage

**Figure 4:**
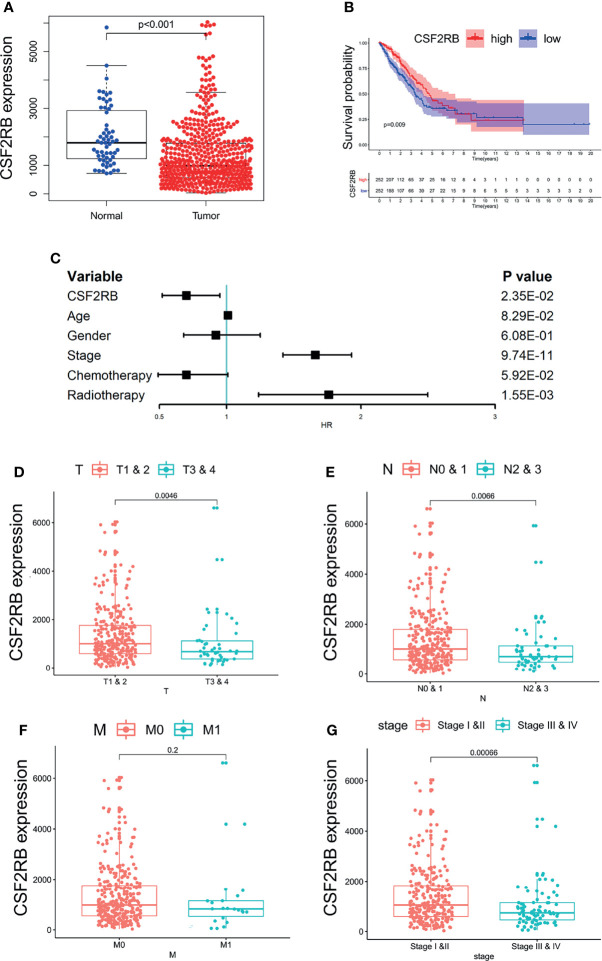
The relationship between the expression of CSF2RB and clinical characteristics. **(A)** The expression of CSF2RB in lung adenocarcinoma tumor tissue and normal tissues. **(B)** A Kaplan–Meier analysis, in which the red line indicates the group with higher expression of this gene, and the blue line indicates the group with lower expression. **(C)** Multivariate cox regression analysis associated with survival. (**D–G)** Relationship between clinical characteristics and the CSF2RB gene.

We used LIMMA package to compare the expression differences of CCR2 and CSF2RB in lung adenocarcinoma tumor tissues and normal tissues. It showed that the expression of CSF2RB in tumor tissues was significantly lower than normal tissues (**Figure 4A**), while the expression of CCR2 remained the same in the two different tissues (**Supplementary Figure 4A**). As a result, CSF2RB became the only candidate in the following study.

To evaluate the prognostic value of CSF2RB, we performed Kaplan–Meier analysis and multivariate COX analysis. The result of Kaplan–Meier analysis showed that the low CSF2RB expression was associated with poor survival in LUAD (**Figure 4B**, p < 0.05). The result of multivariate COX analysis showed that the CSF2RB gene was an independent risk factor for prognosis, regardless of whether the patients received chemotherapy and radiotherapy or not (**Figure 4C**, p < 0.05). Further analysis of the potential correlation between CSF2RB and the clinical characteristics of patients found that the expression of CSF2RB at the age above 65 was notably higher than that of samples at age under 65 and regardless of gender (**Supplementary Figure 4B, C**, p < 0.05). A high expression of CSF2RB was related to early T and N stages (**Figures 4D, E**, p < 0.05). Based on the TNM stage classification, the expression of CSF2RB was significantly higher in early clinical stages (I–II) than in advanced clinical stages (III–IV) (**Figure 4G**, p < 0.05). However, it showed no difference of CSF2RB according to the M stage (**Figure 4F**, p > 0.05). All the above results indicated that CSF2RB could be used as a protective factor, which could also be possibly related to inhibition of lung adenocarcinoma progression.

### GSEA Analysis of CSF2RB Gene Expression and the Correlation Analysis Between CSF2RB and Infiltrated Immune Cells

**Figure 5:**
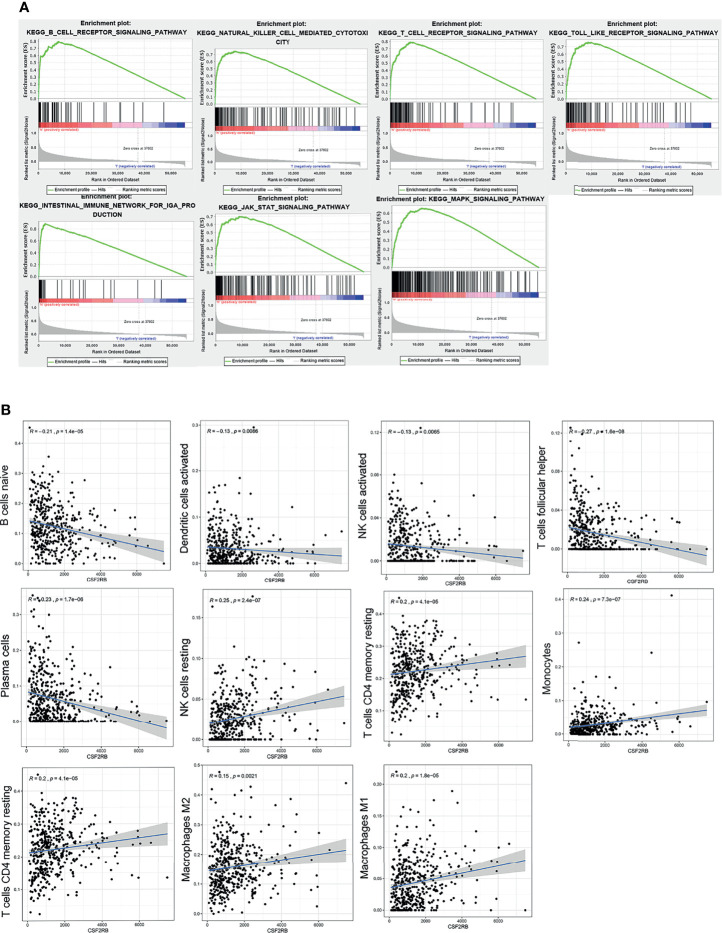
GSEA analysis of CSF2RB gene. **(A)** Immune activation processes were enriched in the CSF2RB high expression group of LUAD. The JAK/STAT and MAPK signaling pathways were enriched in the CSF2RB high-expression group of LUAD. **(B)** The correlation analysis between CSF2RB and infiltrated immune cells.

The whole transcriptome of all tumor samples was used for GSEA analysis. When CSF2RB was highly expressed, various immune-related pathways were also enriched, including natural killer cell-mediated cytotoxicity, intestinal immune network for IgA production, T-cell receptor signaling pathway, Toll-like receptor signaling pathway, B-cell receptor signaling pathway, and CSF2RB which affects the cell cycle and proliferation *via* the JAK/STAT and MAPK signaling pathways (**Figure 5A**).

To better understand the role of CSF2RB in the tumor microenvironment of LUAD, first we used the CIBERSORT algorithm to assess the differences of LUAD patients in 22 TIIC subgroups according to the expression level of CSF2RB. It showed that there was a significant increase in abnormal immune cell infiltrations and heterogeneities. The proportion of naive B cells, plasma cells, follicular helper T cells (Tfh cells), and activated NK cells was high in the CSF2RB low-expression group, while the proportion of resting memory CD4 T cells, resting NK cells, monocytes, M1 macrophages, and M2 macrophages was high in the CSF2RB high-expression group (**Supplementary Figure 5**).

Then we further analyzed the correlation between CSF2RB and each infiltrated immune cell by using the Pearson correlation analysis method. The expression level of CSF2RB was positively correlated with the infiltration of M1 macrophages, M2 macrophages, monocytes, resting NK cells, activated memory CD4 T cells, and T cells CD4 memory resting. Moreover, it was negatively correlated with the level of infiltration of naive B cells, activated dendritic cells, activated NK cells, plasma cells, and T cells follicular helper (**Figure 5B**). These findings suggested that CSF2RB played an important role in regulating the immune cell infiltration in the TME.

### Validate CSF2RB Gene Expression in Lung Cancer Tissues

**Figure 6:**
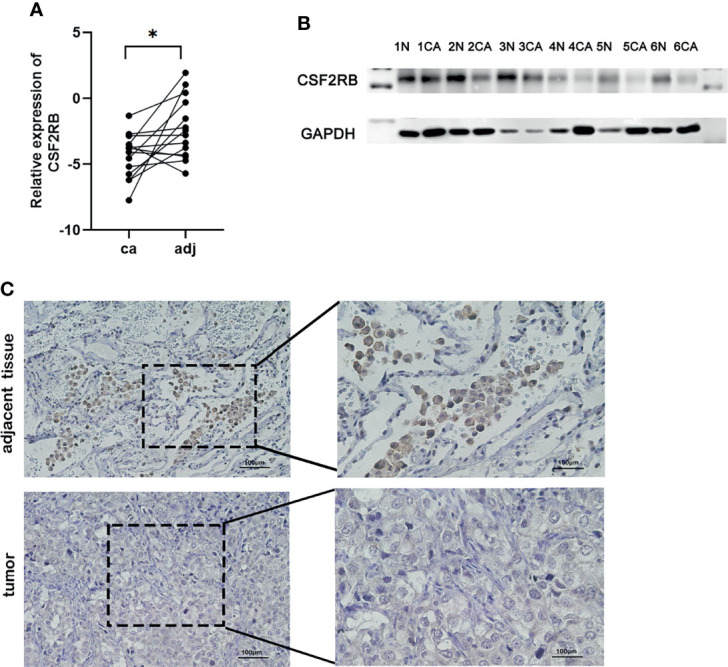
Validate the expression of CSF2RB in lung adenocarcinoma cells. **(A)** RT-qPCR of CSF2RB. Paired sample expression analyses for the CSF2RB gene. *p < 0.05 **(B)** CSF2RB expression in LUAD tissues and adjacent normal tissues assayed by Western blot. **(C)** CSF2RB expression in LUAD tissues and adjacent normal tissues assayed by IHC (×200) and (×400). CA, cancer tissue; N, para-cancer tissue.

To confirm the expression of CSF2RB in lung adenocarcinoma cells, we collected lung adenocarcinoma tissues and para-cancerous tissues from the Tianjin Cancer Hospital and Institute. All selected patients received surgery without neoadjuvant therapy, such as chemotherapy, radiotherapy, and immunotherapy. The clinical data of patients can be obtained in **Supplementary Tables 1** **–** **3**. Then, RT-qPCR assays were performed in 14 patients with the cancer tissues and adjacent normal tissues. Similar to previous findings, CSF2RB expression was reduced in cancer tissues compared with the adjacent normal tissues (**Figure 6A**, p < 0.05). Western blot analysis also confirmed the decrease in CSF2RB protein expression in 6 cancerous tissues than in tumor-adjacent tissues (**Figure 6B**). To further verify this finding, we detected CSF2RB protein expression in 20 pairs of matched lung adenocarcinoma and tumor-adjacent tissues by using immunohistochemistry. CSF2RB had a higher positive expression rate in para-cancerous tissues (100%, 20/20) than those in cancerous tissues (0%, 0/20) (**Figure 6C**). Taken together, we could demonstrate that CSF2RB would be used as an important negative regulator of lung cancer progression.

## Discussion

The incidence of lung cancer has kept increasing in recent years. Surgery, chemotherapy, and radiotherapy are still the main treatments for lung cancer patients, but barely 30% of patients are suitable for surgery by the time of diagnosis. The traditional chemotherapy regimen has limited efficacy, in which the 5-year survival rate is still less than 15%. Meanwhile, the side effects of radiotherapy are relatively large, which limits its application. Therefore, it is necessary to find new targets to improve the current treatment status (22).

In our study, we found that CSF2RB is an important biomarker in LUAD, which may act as a tumor-suppressor gene to inhibit the growth, proliferation, and differentiation of lung adenocarcinoma. Besides, it can also be used as an independent prognostic biomarker. We found that the expression of CSF2RB mRNA in lung adenocarcinoma tissues was significantly lower than in adjacent tissues. Moreover, the low expression of CSF2RB was closely related to the late clinical pathological stages and poor prognosis of lung adenocarcinoma patients, which enabled CSF2RB to serve as an independent prognostic factor. CSF2RB is the shared subunit βc of interleukin 3 (IL3), GM-CSF, and IL5 receptor (21). GM-CSF, IL3, CSF2, and IL5 are important regulators of inflammation and immune suppression within the tumor microenvironment (23). They can produce proliferation and survival signals for many malignant tumors, such as leukemia, small cell lung cancer, melanoma, breast cancer, and prostate cancer (24, 25). Hong et al. found that the transcription factor RUNX1 could bind to the CSF2RB promoter to increase CSF2RB transcription, effectively reducing cell viability, invasion, metastasis, and angiogenesis and promoting neuroblastoma apoptosis *in vitro* and *in vivo* (24). Kao et al. found that CBAP interacted with CSF2RB and induced apoptosis through mitochondrial dysfunction (26). These studies demonstrate that the CSF2RB gene played an important regulatory role in the process of cell growth, proliferation, invasion, and metastasis.

Furthermore, CSF2RB correlated with immune infiltrates in lung adenocarcinoma. The result of the connection between TIICs and CSF2RB suggested that CSF2RB may be involved in regulating the infiltration of immune cells. Moreover, the GSEA result showed that CSF2RB affects the cell cycle and proliferation *via* the JAK/STAT and MAPK and other immune-related signaling pathways. Rummelt et al. found that reactivation of the CSF2RB–STAT5 pathway was the reason for the acute myeloid leukemia resistance to therapeutic FLT3 inhibitors (27). Zsiros et al. showed that the endocytosis of CSF2RB played an important role in regulating the signal transduction of mesothelial-macrophage transformation. In mesenteric mesothelial cells, CSF2RB was internalized in the pit and transmitted to the early endosomes. STAT5A was phosphorylated by JAK-2 and then translocated to the nucleus. When the endocytosis of CSF2RB was inhibited by DATORE, STAT5A did not undergo phosphorylation, confirming that the internalization of receptor β was indispensable for signal transduction (26). CSF2RB mutations can result in ligand-independent activation *in vitro*, according to both spontaneous transformation (22, 28) and random mutation (28, 29). Watanabe-Smith et al. found that CSF2RB mutations in leukemia patients promoted factor-independent growth, receptor phosphorylation and accumulation, and activation of the structural JAK/STAT pathway (21). In Suzuki’s study, CSF2RB mutations almost completely affected the maturation and function of alveolar macrophages leading to the disorder of the degradation of intracellular surface-active substances and the deposition of phospholipids and lipoproteins, which in turn led to severe respiratory insufficiency (30, 31). These studies prove that the CSF2RB gene participates in various regulatory pathways in the process of tumor occurrence and development and plays an important role. It may be used as a therapeutic target to inhibit tumor progression in the future.

During the development of lung adenocarcinoma, tumor cells interacted with TME to improve tumor invasion and metastasis. Immune cells are one of the most important regulatory components in the TME. They were at the center of a series of sequential processes from non-resolving inflammation to tumor metastasis. Exploring the interaction between TME and tumor cells from the perspective of research has gradually become a research hotspot in recent years. Immune and stromal scores were substantially linked with the clinical stage of the patients, according to our findings. Patients with lower TNMs and clinical stage had higher immune and stroma scores and a higher survival rate. With the development of bioinformatics based on tumor microarray sequencing, researchers started to use the ESTIMATE algorithm or some other statistical algorithms to explore the infiltration of immune and stromal cells in TME. In lung cancer, Wu et al. found that patients with higher immune and stromal scores had a more favorable overall survival (OS) than those with lower immune and stromal scores (32–35). Peng et al. used multiplex immunofluorescence to demonstrate that patients with high levels of immune cell infiltration had the longest disease-free survival (DFS). Patients with low immune cell infiltration but rich in cancer stem cells and macrophages suffered worst prognosis (36). In breast cancer, it was reported that high levels of immune infiltration were associated with good clinical outcomes (37). A clinical research based on the immune score model was used to predict the prognosis and chemotherapy effects of breast cancer. It was reported that patients who received chemotherapy shared a better survival in the high-score group, regardless of the chemotherapy regimen itself (38).

By analyzing immune-related genes with patient prognosis and clinical stage, this study made it possible to find molecular markers that can be used as cancer prognosis and provided a reliable theoretical basis for future mechanism study. At the same time, we also verified the expression of the CSF2RB gene by using the tumor tissue in our own institute. However, this study also has certain limitations. The function of the CSF2RB gene has not been deeply studied. In the future, we will go deep into the function analysis of this gene and its potential mechanism on lung cancer progression.

## Data Availability Statement

The original contributions presented in the study are included in the article/**Supplementary Material**. Further inquiries can be directed to the corresponding authors.

## Ethics Statement

The studies involving human participants were reviewed and approved by the Human Ethics Committee of Tianjin Medical University. The patients/participants provided their written informed consent to participate in this study.

## Author Contributions

NZ, YY, and LG: design and initiation of the study, quality control of data, data analysis and interpretation, and manuscript preparation and editing. All authors contributed to the article and approved the submitted version.

## Funding

This study was supported by the National Natural Science Foundation of China (Grant Numbers: 81772619 and 81501994), Clinical Trial Project of Tianjin Medical University (Grant Number: 2017kylc008), Tianjin Medical University Cancer Institute and Hospital Clinical Trials (C1711), Wu Jieping Medical Foundation (Grant Number: 320.2730.1886), Bethune Charity Foundation (Grant Numbers: HZB-20190528-18 and HZB-20190528-11), and the National Natural Science Foundation of China, (Grant/Award Numbers: 82002551 and 81772619).

## Conflict of Interest

The authors declare that the research was conducted in the absence of any commercial or financial relationships that could be construed as a potential conflict of interest.

## Publisher’s Note

All claims expressed in this article are solely those of the authors and do not necessarily represent those of their affiliated organizations, or those of the publisher, the editors and the reviewers. Any product that may be evaluated in this article, or claim that may be made by its manufacturer, is not guaranteed or endorsed by the publisher.
