# Supplementary material for: CSF2RB Is a Unique Biomarker and Correlated With Immune Infiltrates in Lung Adenocarcinoma
Source: Front Oncol. 2022 Apr 28;12:822849. doi: 10.3389/fonc.2022.822849 (PMC9096117; doi:10.3389/fonc.2022.822849)
Supplement: Supplementary file 1 [file DataSheet_1.docx]

Introduction for raw data

Because the raw data was large, I have uploaded them to <https://www.jianguoyun.com/>, and have set the sharing scope for any download links to “Anyone”.

1 The R code data download link is

<https://www.jianguoyun.com/p/DQrXhR8Q75OBChjj7pwE>

The name of the folder is “raw data-R code”

2 The raw data of TCGA download link is

<https://www.jianguoyun.com/p/DfRPihUQ75OBChjk7pwE>

The name of the folder is “raw data-TCGA”

3 The raw data download links of figure 7a are <https://www.jianguoyun.com/p/DXvfHA4Q75OBChiR7pwE>

<https://www.jianguoyun.com/p/DcZMNJQQ75OBChia7pwE>

The name of the folder are “raw data-RT-qPCR” and “RT-qPCR Statistical Analysis”

4 The raw data download link of figure 7b is

<https://www.jianguoyun.com/p/DX8zq2EQ75OBChik7pwE>

The name of the folder is “raw data-Western Blot full scans”

5 The raw data download links of figure 7c are

<https://www.jianguoyun.com/p/DWsKDM8Q75OBChiy7pwE>

<https://www.jianguoyun.com/p/DfAw9SQQ75OBChi07pwE>

<https://www.jianguoyun.com/p/DfZuZG4Q75OBChi67pwE>

<https://www.jianguoyun.com/p/DYubPq4Q75OBChjB7pwE>

The names of the folder are “raw data-IHC (1)、（2）、（3）”


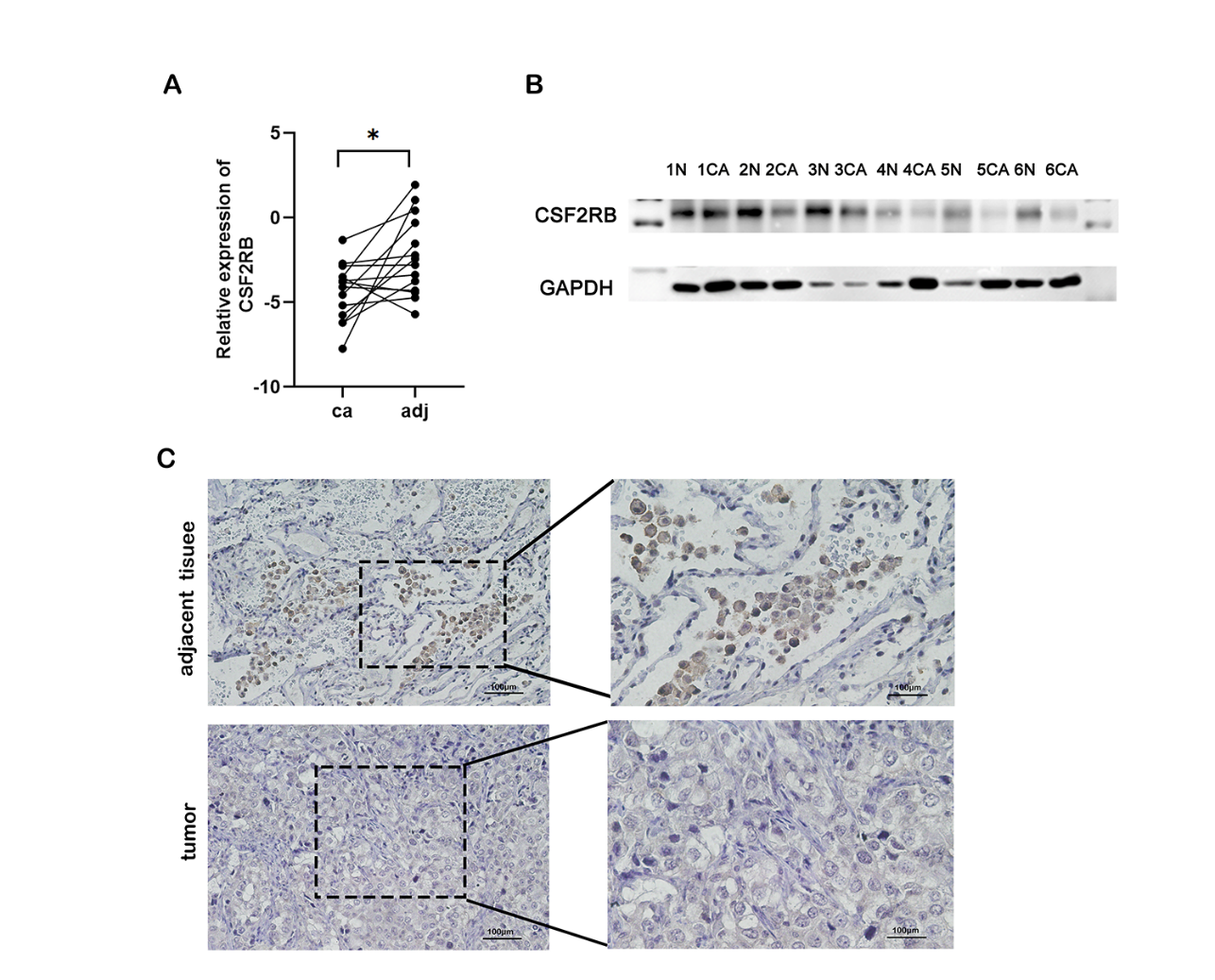
**Figure 7**
