# Supplementary material for: CSF2RB Is a Unique Biomarker and Correlated With Immune Infiltrates in Lung Adenocarcinoma
Source: Front Oncol. 2022 Apr 28;12:822849. doi: 10.3389/fonc.2022.822849 (PMC9096117; doi:10.3389/fonc.2022.822849)
Supplement: Supplementary file 2 [file DataSheet_2.zip › Supplementary Figure legend.docx]

**Supplementary Figure 1.** **The relationship between immune and** **stromal scores and clinical features and prognosis of lung adenocarcinoma. (A)(B)(C)** The correlation between immune scores with clinicopathological features. (**D)(E)(F)** The correlation between stromal scores with clinicopathological features.

**Supplementary Figure 2. Infiltration analysis of 22 types of immune cells. (A)** The distribution of TIIC in each sample (ie 59 adjacent samples and 515 LUAD patients). Each column represents a sample, and each column with a different color and height indicates the abundance ratios of immune cells in this sample. **(B)** the correlation between the various TIICs of LUAD. The value represents the correlation value. Blue represents a positive correlation, and the orange represents a negative correlation.

**Supplementary Figure 3. The relationship between immune cells and clinical features and prognosis of lung adenocarcinoma. (A)** The relationship between immune cells and clinical features. **(B)** The relationship between immune cells and survival rate of lung adenocarcinoma.

**Supplementary Figure 4 (A)**The expression of CCR2 in tumor tissues and normal tissues. **(B) (C)** Relationship between the expression of CSF2RB and gender/M stage.

**Supplementary Figure 5 The differences of LUAD patients in 22 TIIC subgroups.** Horizontal and vertical axes respectively represent TIICs and relative percentages. Green and red colors represent CSF2RB gene low expression sample groups and CSF2RB gene high expression sample groups, respectively. Data were assessed by the Wilcoxon rank-sum test.
