# Supplementary figures and images for: CSF2RB Is a Unique Biomarker and Correlated With Immune Infiltrates in Lung Adenocarcinoma
Source: Front Oncol. 2022 Apr 28;12:822849. doi: 10.3389/fonc.2022.822849 (PMC9096117; doi:10.3389/fonc.2022.822849)

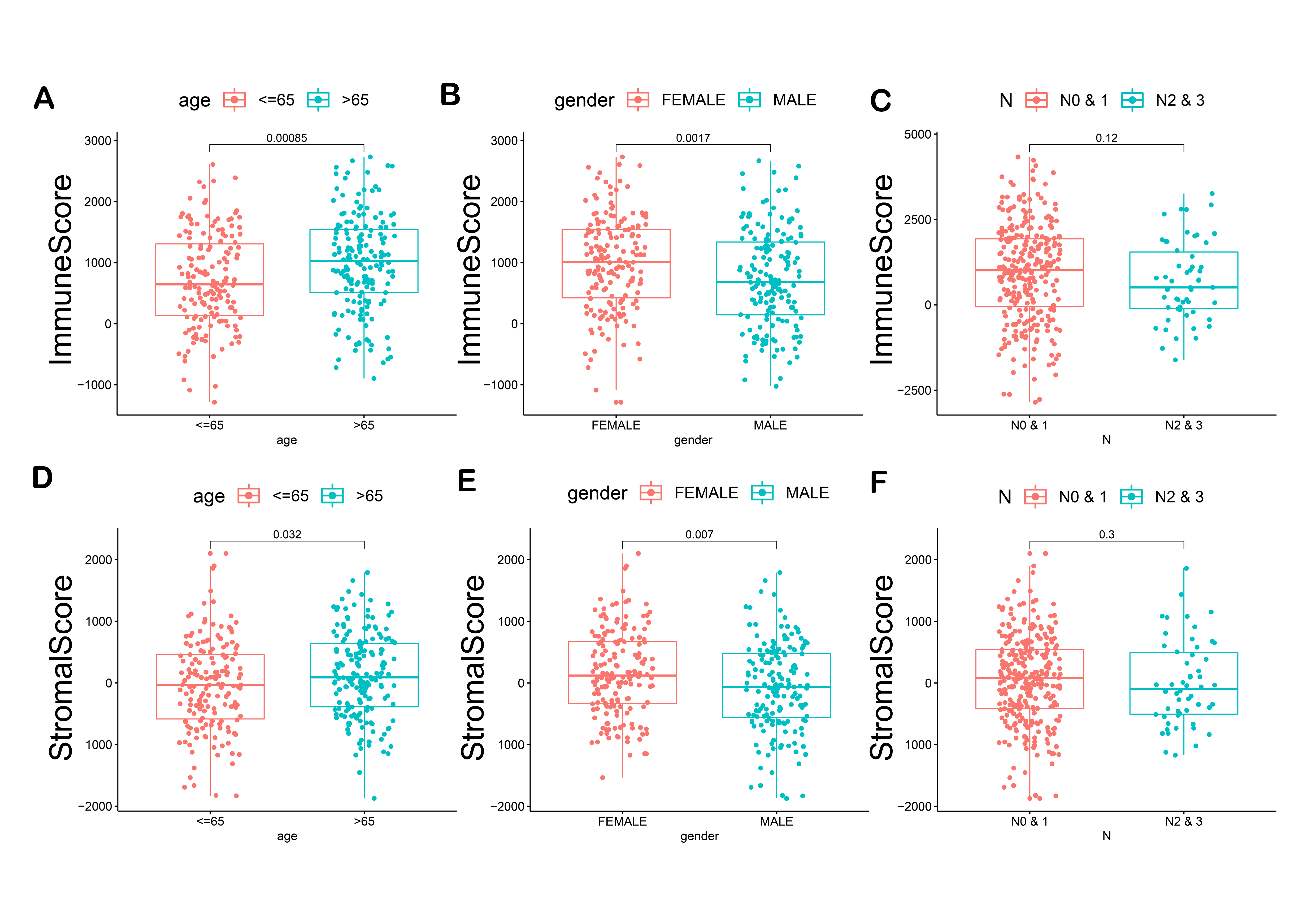

Supplement: Supplementary file 2 [file DataSheet_2.zip › Supplementary Figure 1 .tif]

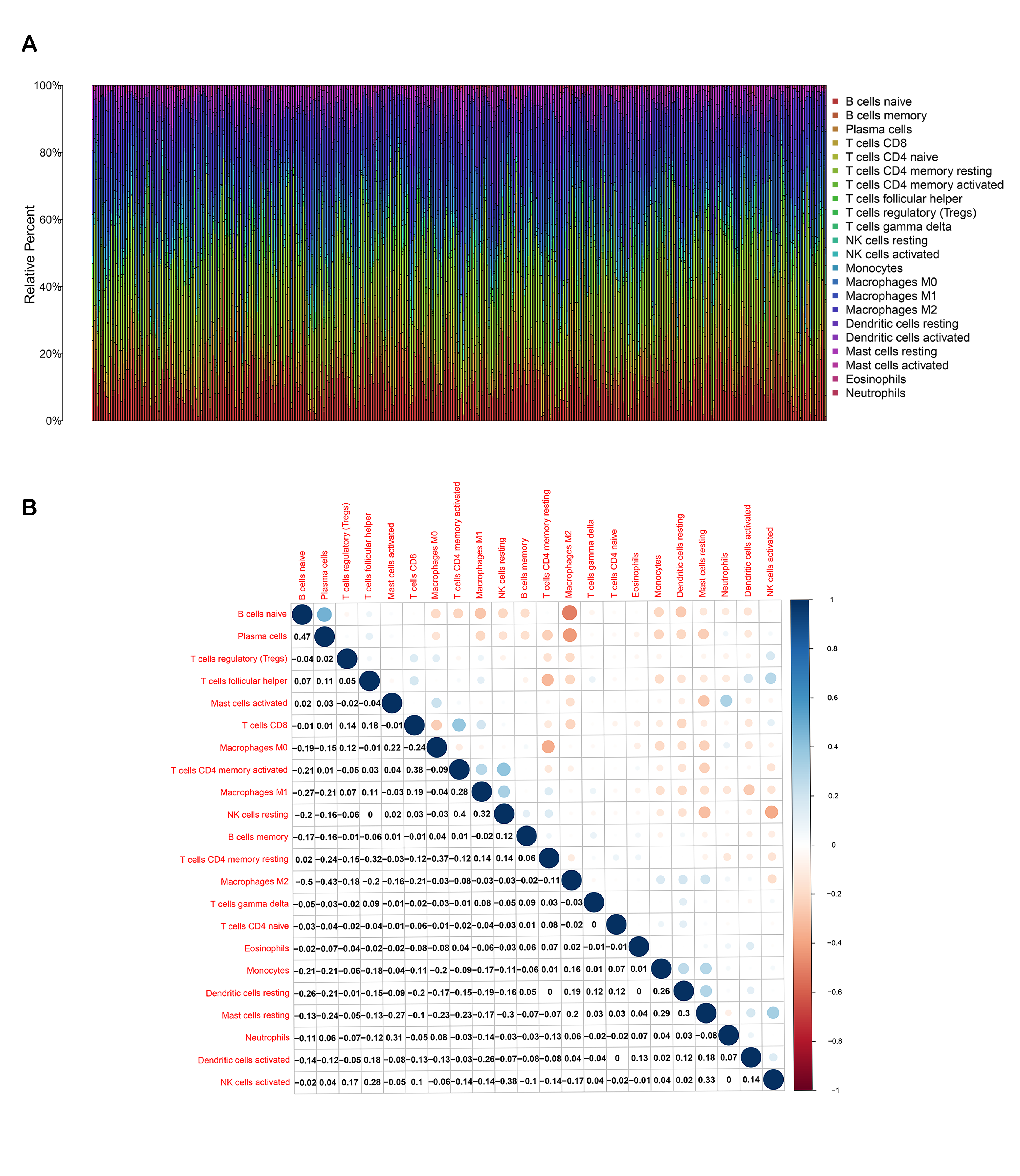

Supplement: Supplementary file 2 [file DataSheet_2.zip › Supplementary Figure 2.tif]

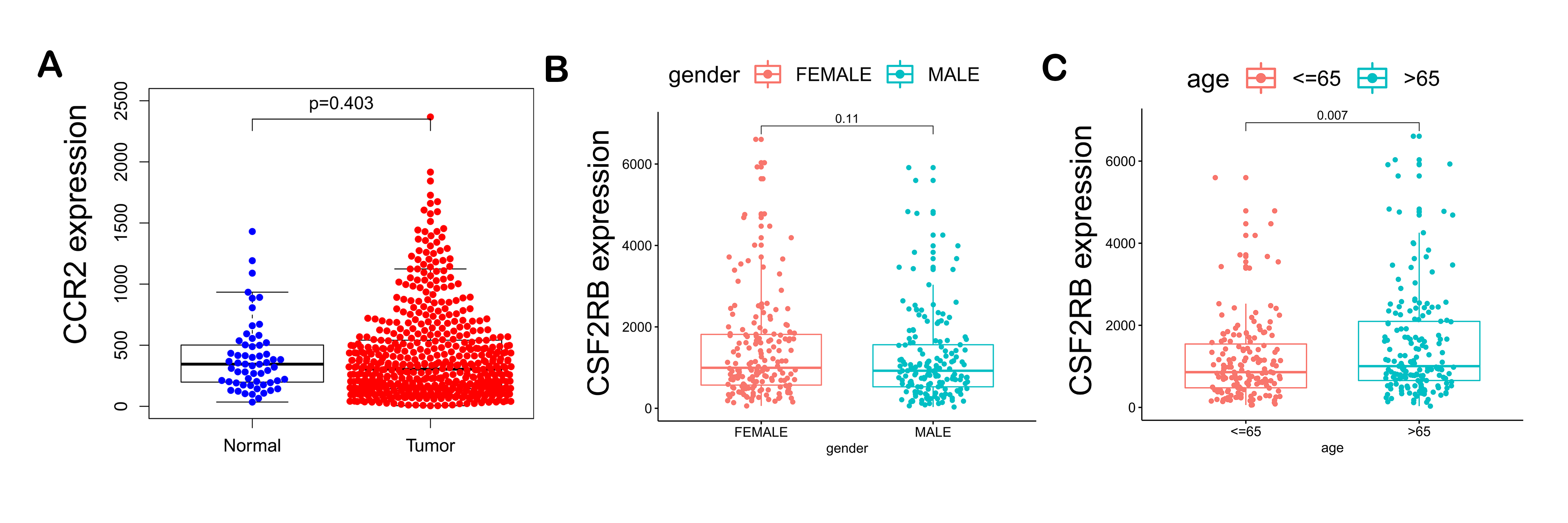

Supplement: Supplementary file 2 [file DataSheet_2.zip › Supplementary Figure 4.tif]

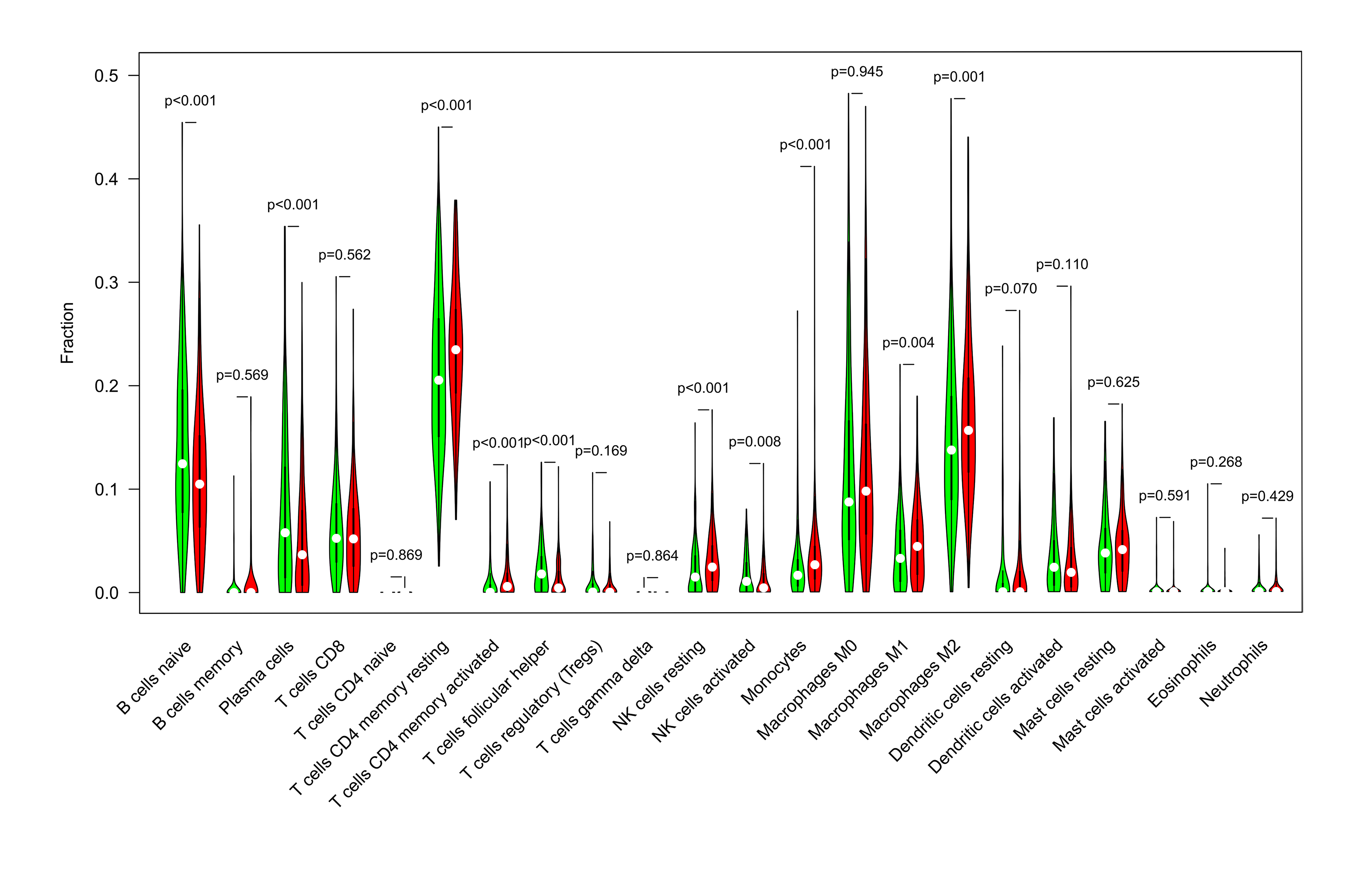

Supplement: Supplementary file 2 [file DataSheet_2.zip › Supplementary Figure 5.tif]
