# Supplementary material for: CSF2RB Is a Unique Biomarker and Correlated With Immune Infiltrates in Lung Adenocarcinoma
Source: Front Oncol. 2022 Apr 28;12:822849. doi: 10.3389/fonc.2022.822849 (PMC9096117; doi:10.3389/fonc.2022.822849)
Supplement: Supplementary file 4 [file Table_2.docx]

**Table S2. The details of subjects for Western Blot**

| **No.** | **Gender** | **Age** | **T** | **N** | **M** | **Stage** | **Neoadjuvant**  **therapy** |
| --- | --- | --- | --- | --- | --- | --- | --- |
| 1 | Female | 55 | T1a |  |  | IA1 | No |
| 2 | Male | 52 | T1a |  |  | IA1 | No |
| 3 | Female | 54 | T1b |  |  | IA2 | No |
| 4 | Female | 72 | T1b |  |  | IA2 | No |
| 5 | Male | 56 | T2 |  |  | IB | No |
| 6 | Female | 35 | T1a |  |  | IA1 | No |
