# Supplementary material for: CSF2RB Is a Unique Biomarker and Correlated With Immune Infiltrates in Lung Adenocarcinoma
Source: Front Oncol. 2022 Apr 28;12:822849. doi: 10.3389/fonc.2022.822849 (PMC9096117; doi:10.3389/fonc.2022.822849)
Supplement: Supplementary file 5 [file Table_3.docx]

**Table S3. The details of subjects for IHE**

| **No.** | **Gender** | **Age** | **T** | **N** | **M** | **Stage** | **Neoadjuvant therapy** |
| --- | --- | --- | --- | --- | --- | --- | --- |
| 1 | Female | 54 | T1c | N2 | M1a | IVA | No |
| 2 | Female | 65 | T1c | N1 | M1a | IVA | No |
| 3 | Female | 55 | T2a | N2 | M1a | IVA | No |
| 4 | Male | 64 | T1c |  | M1a | IVA | No |
| 5 | Male | 64 | T1c | N2 | M1a | IVA | No |
| 6 | Female | 55 | T1b | N2 |  | IIIA | No |
| 7 | Female | 57 | T1c | N2 |  | IIIA | No |
| 8 | Female | 52 | T3 | N2 |  | IIIB | No |
| 9 | Male | 58 | T3 | N2 |  | IIIB | No |
| 10 | Female | 66 | T1c | N1 |  | IIB | No |
| 11 | Female | 45 | T1c | N1 |  | IIB | No |
| 12 | Female | 71 | T1c | N1 |  | IIB | No |
| 13 | Female | 69 | T1C |  |  | II | No |
| 14 | Male | 71 | T1c |  |  | I | No |
| 15 | Male | 67 | T1C |  |  | I | No |
| 16 | Male | 67 | T1C |  |  | I | No |
| 17 | Male | 67 | T1C |  |  | I | No |
| 18 | Male | 56 | T1C |  |  | I | No |
| 19 | Female | 57 | T1C |  |  | I | No |
| 20 | Female | 65 | T1C |  |  | I | No |
